# Design and development of a biorelevant simulated human lung fluid

**DOI:** 10.1016/j.jddst.2018.08.006

**Published:** 2018-10

**Authors:** Mireille Hassoun, Paul G. Royall, Mark Parry, Richard D. Harvey, Ben Forbes

**Affiliations:** a King's College London, Institute of Pharmaceutical Science, London, SE1 9NH, UK; b Intertek-Melbourn Scientific Limited, Melbourn, SG8 6DN, UK; c Institute of Pharmacy, Martin-Luther-Universität Halle-Wittenberg, 06108, Halle (Saale), Germany

**Keywords:** Respiratory, Simulated lung fluid (SLF), Surfactant, Dissolution, Solubility, Inhalation biopharmaceutics

## Abstract

Biorelevant fluids are required to enable meaningful *in vitro* experimental determinations of the biopharmaceutical properties of inhaled medicines, e.g. drug solubility, particle dissolution, cellular uptake. Our aim was to develop a biorelevant simulated lung fluid (SLF) with a well-defined composition and evidence-based directions for use. The SLF contained dipalmitoylphosphotidylcholine, dipalmitoylphosphatidylglycerol, cholesterol, albumin, IgG, transferrin and antioxidants. Freshly made SLF had pH 7.2, viscosity 1.138 × 10^−3^ Pa s, conductivity 14.5 mS/m, surface tension 54.9 mN/m and density 0.999 g/cm^3^. Colour, surface tension and conductivity were the most sensitive indicators of product deterioration. The simulant was stable for 24 h and 48 h at 37 °C and 21 °C, respectively, (in-use stability) and for 14 days when stored in a refrigerator (storage stability). To extend stability, the SLF was vacuum freeze-dried in batches to produce lyophilised powder that can be reconstituted readily when needed at the point of use. In conclusion, we have reported the composition and manufacture of a biorelevant, synthetic SLF, provided a detailed physico-chemical characterisation and recommendations for how to store and use a product that can be used to generate experimental data to provide inputs to computational models that predict drug bioavailability in the lungs.

## Graphical abstract

**Figure:**
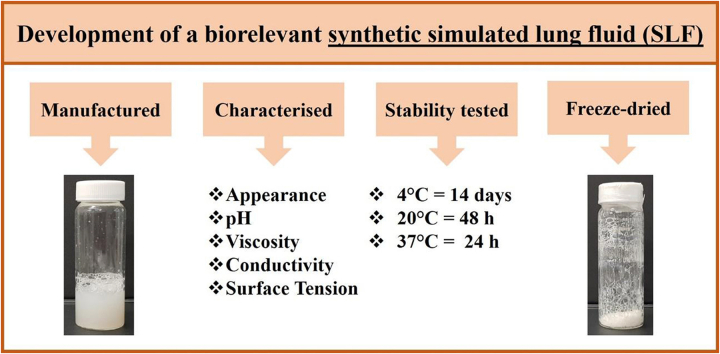

## Abbreviation section

RTLF: Respiratory Tract Lining Fluid
SLF: Simulated Lung Fluid
DPPC: 1,2-dipalmitoyl-*sn*-glycero-3-phosphocholine
DPPG: 1,2-dipalmitoyl-*sn*-glycero-3-phospho-(1′-*rac*-glycerol) sodium salt
IgG: Immunoglobulin G
HBSS: Hank's Balanced Salt Solution
TLC: Thin Layer Chromatography
TGA: Thermogravimetric Analysis

## 1 Introduction

The factors that govern respiratory and systemic exposure to drugs delivered as inhaled medicines define their ‘inhalation biopharmaceutics’ [1]. The major factors determining the bioavailability of drugs delivered as inhaled medicines are lung dose and aerodynamic particle size distribution, which determines regional deposition [2]. Following deposition, the interactions of aerosol particles or drug with competing lung clearance mechanisms determine (i) drug bioavailability in the lungs; (ii) the rate and extent of absorption to the systemic circulation [3]. Drug solubility is a key consideration in the development of inhaled medicines, including drug design/discovery [4,5], formulation [6] and toxicokinetics [7]. The importance of solubility and dissolution in predicting the pharmacokinetics of some orally inhaled drug products has been demonstrated convincingly [8] and particle-lung fluid interactions have been suggested to impact on inhaled drug delivery [9]. For example, excipients such as glycerol in licenced inhaled medicines have been shown to influence aerosol particle dissolution [10,11] and interactions between inhaled nanomaterials and lung fluid have been investigated [12,13]. Study of all these phenomena require biorelevant fluids in which to make meaningful *in vitro* experimental measurements.

Since the first biological destination of inhaled medicinal aerosols is the respiratory tract lining fluid (RTLF) in which they deposit, it is surprising how little is published about models of RTLF in which to study interactions with particles, e.g. requirements for fluid composition or critical attributes. A variety of approaches have been adopted to simulate RTLF, including use of surfactant solutions, e.g. Tween 80 or sodium dodecyl sulphate in phosphate buffered saline [8,10], phospholipid-containing fluids [14,15] or diluted surfactant replacement products, such as Alveofact, Exosurf, Curosurf or Survanta [16,17]. Recently, a synthetic simulated lung fluid (SLF) based on human lung fluid composition has been characterised in terms of its vesicular structure and particle size, surface pressure and assessed for biocompatibility with A549 alveolar epithelial cells [18]. A similar SLF has been reported for applications in environmental toxicology [19], but here we provide a more detailed validation and characterisation of a ‘base’ SLF that reflects human lung fluid composition and can be incorporated into *in vitro* experimental models or adapted for specific applications in pharmaceutical science, e.g. if particular surfactant proteins, specific metabolic activities or model inflammatory disease states are of interest.

If a simulant is to prove useful, it must be readily available, convenient, economic and have well defined conditions for storage and use. For a complex aqueous fluid with components susceptible to chemical degradation, physical instability or microbial spoilage, freeze-drying provides an excellent means of preservation. Desiccation will protect the SLF since it minimises chemical reactions, e.g. the rate of lipid hydrolysis during storage [20], and deters microbiological growth. Accordingly, freeze-drying the SLF would allow batch manufacture in a form that has an extended use-by date and is easily handled and transported for reconstitution at its place and time of use [21,22].

This study provides a complete description of the design and manufacture of the biorelevant SLF of Kumar and co-workers [18] and reports a suite of physicochemical parameters that characterise the product and can be used as sensitive indicators of SLF stability. The stability of SLF was investigated to define appropriate conditions for its storage and use. We also investigated freeze-drying the lung fluid simulant to produce a lyophilised powder with long term stability that can be reconstituted when required at the point of use.

## 2 Materials and methods

The 25 mg/mL stock solutions of 1,2-dipalmitoyl-*sn*-glycero-3-phosphocholine (DPPC) and 1,2-dipalmitoyl-*sn*-glycero-3-phospho-(1′-*rac*-glycerol) sodium salt (DPPG), both >99% purity, were obtained from Avanti Polar Lipids, Inc. (Alabama, USA). Reagent-grade purified human immunoglobulin (IgG), lyophilised human serum albumin, Bioreagent-grade transferrin, cholesterol, ascorbate, urate, certified reference material-grade glutathione and BioReagent grade gentamicin solution were supplied by Sigma-Aldrich Company Limited (Dorset, UK). Hanks' Balanced Salt Solution (HBSS), phenol red-free, was also supplied by Sigma-Aldrich and consisted of: 0.19 g/L calcium chloride dihydrate, 0.09 g/L magnesium sulphate anhydrous, 0.40 g/L potassium chloride, 0.06 g/L potassium phosphate, 0.35 g/L sodium bicarbonate, 8.00 g/L sodium chloride, 0.05 g/L sodium phosphate dibasic, and 1.00 g/L d-Glucose. HPLC-grade chloroform and methanol was supplied by Fischer Chemicals (Loughborough, UK). 25% ammonium hydroxide, sodium chloride and 2 M hydrochloric acid solutions were obtained from Sigma-Aldrich Company Limited (Dorset, UK).

### 2.1 Preparation of SLF

**Fig. 1:**
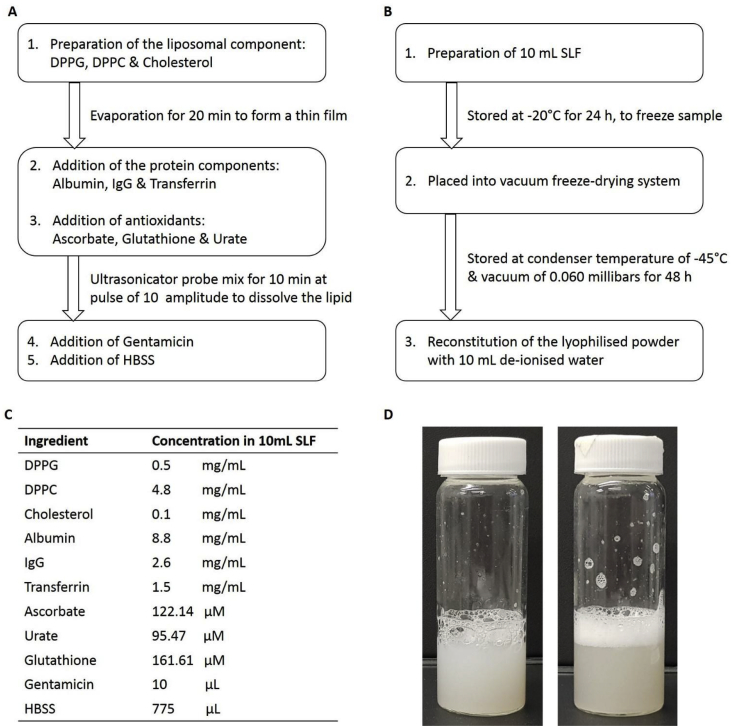
A) Manufacturing steps of simulated lung fluid (SLF), B) Freeze-drying and reconstitution steps of SLF, C) Composition of SLF and D) Images of the original and the reconstituted SLF.

SLF consisted of the key components found in healthy human RTLF, the major soluble proteins, the abundant lipids and the antioxidants that were identified in a study by Bicer [23]. The proteins were albumin, IgG and transferrin, and the lipids were DPPC, DPPG and cholesterol. The preparation method was optimised into two stages, with the manufacture of a liposomal dispersion followed by addition of the proteins (Fig. 1). To prepare the liposomal component, 1.92 mL DPPC and 0.2 mL DPPG, from 25 mg/mL stock solutions in chloroform were combined in a bijou bottle, with 5 μL of cholesterol from a 200 mg/mL stock solution in chloroform also added. The mixture was stirred gently and the chloroform evaporated under a stream of nitrogen gas for 30 min (sufficient to ensure that the lipid film was solvent free) to produce a thin film of lipids. The proteins were added to the lipid film in aliquots of aqueous stock solutions: 4 mL of albumin (88 mg/mL), 4 mL of IgG (26 mg/mL) and 1 mL of transferrin (15 mg/mL). To represent lung antioxidant levels, 88.5 μL of the following antioxidant stock solutions were added: 10 mM ascorbate, 10 mM glutathione, and 5 mM urate in the HPLC-grade water. The mixture was vortexed for 5 min. Using an ultrasonicator/probe for 10 min at a pulse of 10 amplitude, the lipids were dispersed into the solution in the form of polydisperse multilamellar liposomes. Finally, 10 μL of 50 mg/mL gentamicin was added, followed by 775 μL of HBSS under gentle agitation.

### 2.2 Characterisation of SLF

The SLF was characterised in this study in terms of its pH, density, viscosity, conductivity and surface tension. The pH was measured using a pH probe (pH level 2 InoLab, WTW, Germany). The density of SLF was measured using a density meter (DMA 35, Anton Paar, UK) whereby the inverted capillary cell was filled with approximately 5 mL of SLF. Distilled water was used as the reference. The viscosity was measured using the Automated Micro Viscometer (AMVn 320, Anton Paar, UK). A 1.6 mm diameter capillary, containing a 1.5 mm diameter ball was filled with 400 μL SLF, sealed with a luer cap and measurements made with the capillary tilted at 50°, 60° and 70°. The conductivity of SLF was measured using a conductivity probe and meter (Jenway A520, Cole-Parmer, UK), calibrated using 0.01 M potassium chloride solution, providing a value of 1413 ± 1 μS/cm. The surface tension of SLF was measured using a torsion balance (Model OS, TBS, UK), using distilled water as the reference sample which gave a surface tension value of 72.5 ± 0.5 mN/m. A 4 cm circumference platinum ring was immersed approximately 0.5 cm into SLF ring and the force required for withdrawal of the platinum ring from the surface of the fluid was recorded as the surface tension. All measurements were conducted in triplicate at ambient temperature.

### 2.3 Freeze-drying of SLF

SLF, 10 mL solution, was prepared in a SCHOTT tubular glass freeze drying vial. Samples were frozen overnight at −20 °C and then placed into a vacuum freeze-drying system (Lyotrap, LTE Scientific Ltd, UK) with condenser temperature −45 °C and a vacuum of 0.060 mbars for 48 h. Thermogravimetric analysis (Discovery TGA, TA Instruments, UK) was used to analyse the residual moisture content in the freeze-dried SLF powder, heating the sample at a heating rate of 10 °C/min to a maximum of 200 °C. The powder was reconstituted with 10 mL de-ionised water and compared to fresh SLF using the physico-chemical parameters described in section 2.2.

### 2.4 Stability of SLF

SLF stability was determined for samples stored in the fridge (at 4 °C), at room temperature (20 °C) and at human physiological temperature (37 °C). The stability was assessed by analysing pH, viscosity, conductivity and surface tension after their storage for 0, 1, 2, 3, 4, 7, 14 and 28 days using the methods described above. The appearance and colour of SLF were also analysed visually and via measurements of the mean grey value, using the Image J software (Java 1.8.0–25, version 1.51p). Using the software, the images captured were first converted to ‘8-bit’ prior to obtaining the mean grey value. All data were recorded as the mean of three measurements ± SD.

Lipid degradation was evaluated using one-dimension thin layer chromatography (TLC). The lipid components were extracted from the SLF fluid, using an adapted version of the method detailed by Bligh and Dyer [24]. Briefly, 1 mL SLF sample was centrifuged at 13,000 rpm for 10 min to sediment out the lipids and proteins. To the pellet obtained, 1 mL of a mix consisting of chloroform: methanol [2:1] and 0.26 mL, was added and mixed to form a single phase. The sample was left to incubate with gentle agitation for 90 min at 37 °C. Acidified saline (150 mM sodium chloride adjusted to pH 2 with hydrochloric acid), 0.5 mL, was added and vortex mixed for 5 min followed by centrifugation at 2000 rpm for 15 min (Biofuge Pico, Jencons-PLS Scientific, UK). The aqueous phase was removed and 0.25 mL methanol and 0.25 mL acidified saline was added to the remaining organic phase. This was vortex mixed and centrifuged again at 2000 rpm for 15 min, then the aqueous layer was removed and the organic solvent evaporated to concentrate the lipids.

To analyse the isolated lipids by TLC, the sample was spotted approximately 1 cm from the bottom of a plain silica gel 60 TLC plate. The plate was transferred to a glass TLC tank containing 1 cm depth of the mobile phase, consisting of chloroform, methanol and 25% ammonium hydroxide solution at a ratio of 65:25:10. When the mobile phase reached three-quarters way up the plate the lipids were visualised as yellow spots using potassium permanganate stain. The retardation factor (RF) was calculated as the distance travelled by centre of the spot divided by the distance travelled by the solvent front. A calibration between spot intensity (using the mean grey value) and concentration of DPPC and DPPG was established and used to estimate the content of lipid in the SLF.

### 2.5 Statistical analysis

The physicochemical properties were derived from three independent batches; the data for the stability study was for 3 vials from a single batch of SLF. One-way ANOVA or paired sample T-test was applied, using the IBM SPSS version 24 software, to determine the statistical significance of results. Data was statistically significant when p ≤ 0.05.

## 3 Results and discussion

### 3.1 Composition and characterisation of SLF

The composition of the simulated lung fluid was identical to that reported previously [18] and was designed to provide a simulant that trades off the biorelevance of featuring the full complexity of lung lining fluid with the pragmatic need for a simulant that can be manufactured economically, reproducibly and is fit for the majority of applications in inhaled medicines biopharmaceutics. Although DPPC, which contributes approximately 80% w/v of surfactant phospholipids, is often used as a sole representative phospholipid component in model lung fluids, for physiological relevance other lipid components were incorporated including DPPG which represents approximately 10% of total phospholipids, and cholesterol which accounts for 5–10% lipids and has been shown to have a stabilising role in bilayer structures. Although lung fluid contains a mixture of saturated and unsaturated lipid, the unsaturated species were avoided as being more susceptible to oxidation. Urea corrected total protein concentration measured in human lavage samples is in the range 17.9 ± 8.6 mg/mL [23], but due to the limited commercial availability and consistency of some human protein components, e.g. SP-A, SP-B, SP-C, IgA and A1TA, the total proteins used in the preparation of SLF was 12.9 mg/mL. Biorelevant concentrations of antioxidants were included to promote the reported biocompatibility of the SLF with respiratory epithelial cells [18].

Appearance and four physicochemical properties were used to characterise SLF: colour, pH, conductivity, viscosity and surface tension (Table 1). The pH of SLF was pH 7.2, buffered by the use of HBSS as a base medium. The pH the human respiratory tract is subject to some variability, e.g. pH 6.3 (range 5.2–8.1) in the nasal cavity [25], pH 6.9–9.0 in the trachea [26] and suggested to be more acidic in inflammation and infection [26]. The essential inorganic ions of the HBSS provide a physiological salt composition, tonicity (280 mOsM) and are responsible for the high conductivity in comparison to de-ionised water matching the properties of lung lining fluid in healthy subjects [27]. The surface tension of SLF, 55 mN/m, was lower than that of water which was measured to be 72 mN/m. DPPC is the most surface-active molecule in the pulmonary surfactant mix and significantly reduces surface tension [28]. Pulmonary surfactants in the epithelial lung lining form a lipid-rich film with a significantly reduced surface tension from 70 mN/m at the upper airways to near 0 mN/m at the alveoli [29,30]. The absence of surfactant proteins in SLF (particularly surfactant protein B), which are present in epithelial lung lining fluid result in the higher surface tension value than would otherwise be anticipated for a lung fluid simulant [29,31]. Surfactant proteins also play a role in fluid viscosity, whereby surfactant protein-C (SP-C) interacts with the lipids to produce high surface viscosity [32]. Despite the absence of SP-C in SLF, the viscosity of SLF was higher than that of simple aqueous solutions, which was attributed to the presence of protein macromolecules and complex lipids in their physiological concentrations. The absence of specialist surfactant proteins (SP) limits the application of the SLF for some specialist immunological applications, e.g. where SP A and D are important in host-defence or biophysical studies where SP C and B have important roles in the complex membrane network in the lung lining fluid sub-phase.

**Table 1:** Physicochemical properties of simulated lung fluid (SLF) when freshly manufactured and when reconstituted after freeze-drying. All measurements made at 25 °C, data represent mean ± SD (n = 3).

Physicochemical property	SLF before freeze-dried	SLF after freeze-dried
Appearance [mean grey value]	168.6 ± 0.4	168.6 ± 0.1
pH	7.2 ± 0.0^a^	7.7 ± 0.1^a^
Conductivity [mS/m]	14.5 ± 0.1	14.6 ± 0.2
Viscosity [Pa.s x 10^−3^]	1.138 ± 0.008	1.111 ± 0.015
Surface Tension [mN/m]	54.9 ± 0.3	55.6 ± 0.7

a Difference is significant (T-test, p ≤ 0.05).

### 3.2 Freeze-dried SLF

For freeze drying to be successful, a dry fluffy lyophilised powder should be produced which can easily be reconstituted to possess physiochemical properties that match the original sample [33]. Without addition of cryoprotectants, lyophilised liposomal powders are often compact, clumpy and difficult to reconstitute [20,34,35]. SLF was freeze-dried successfully without the need for additional formulation components to act as cryoprotectants; the powder produced was fluffy and easy to reconstitute with water to restore the appearance and physicochemical properties of the SLF (Table 1). The reconstituted freeze-dried formulation was identical to the freshly made solution except for a small statistically significant increase in pH (one-way ANOVA, p ≤ 0.05), which can be readily corrected. Although water is predominantly neutral in pH, the use of different grades for reconstitution, e.g. distilled water, de-ionised water, HPLC-grade water or ultrapure water, may result in variations in pH [36].

While freeze-drying can improve the storage stability of liposomal formulations, it has been recognised that the process can also cause damage to the liposome structures which are exposed to stress conditions, e.g. excessive dryness, which can pierce the liposomes or predispose them to aggregation [20,37,38]. TGA indicated that 4.42 ± 0.15% moisture was retained in the lyophilised powder produced. In combination, the avoidance of excessive dryness, the likelihood that formulation components provide a degree of cryoprotection and the polydisperse multivesicular nature of the liposomes rendered the SLF resistant to destabilising stress factors under the freeze-drying process parameters employed, i.e. freezing rate, freezing temperature and processing time.

### 3.3 Stability of SLF

**Fig. 2:**
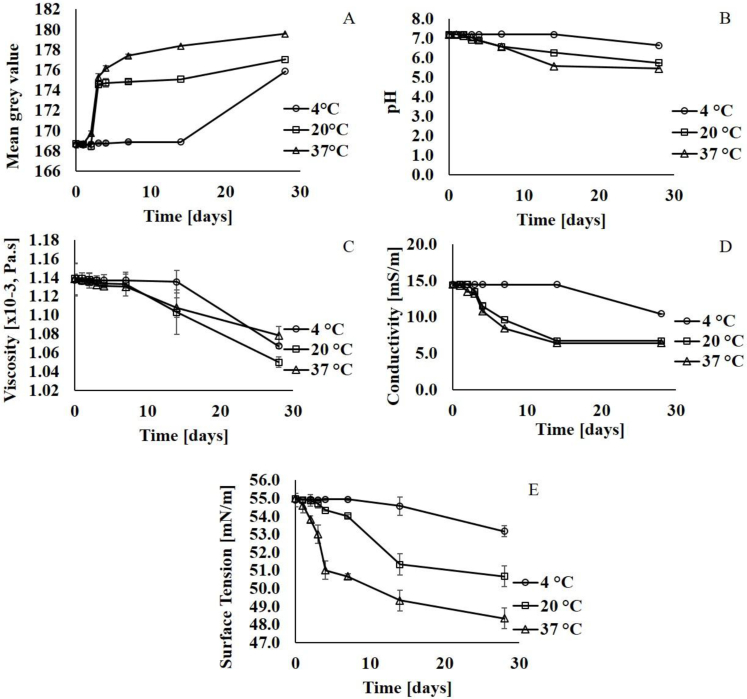
Stability assessment of simulated lung fluid (SLF) after its storage at 4, 20 and 37 °C for 0, 1, 2, 3, 4, 7, 14 and 28 days: A) Colour, B) pH, C) viscosity, D) conductivity, and E) surface tension. Data expressed as mean ± SD (n = 3).

Preliminary studies showed that appearance and four physicochemical properties were stability-indicating; these were used to evaluate SLF over 28 days at three temperatures, 4, 20 and 37 °C (Fig. 2). The assays were congruous in detecting the conditions and time at which SLF began to degrade and in profiling temporal stability at each storage temperature. Although the use of dynamic light scattering was considered as a means to study any changes in the polydispersity and the size distribution of the structures in SLF, in previous studies [18] the extent of background scattering by the multi-molecular protein aggregates present in the fluid has complicated interpretation of results and we found it was not suitable as a stability test.

In terms of appearance, SLF was peach coloured when freshly made. This was quantified using Image J as a mean grey value on a scale where 0 corresponds to black and a value of 255 corresponds to white (Fig. 2A). SLF lightened in colour visibly, becoming whiter by day 2, with the mean grey value rising from 168.5 ± 0.4 to 174.5 ± 0.1 at 20 °C and 169.7 ± 0.3 to 175.3 ± 0.3 at 37 °C. At 4 °C, the mean grey value was unchanged at day 14 and the only increased from 168.9 ± 0.3 to 175.9 ± 0.3 at day 28. The pH and viscosity of the SLF samples stored at 20 and 37 °C also began to decrease after day 2 of storage (Fig. 2B and C). Conductivity and surface tension were affected similarly (Fig. 2D and E), at 37 °C there was a reduction of surface tension from 54.6 ± 0.5 mN/m to 53.0 ± 0.2 mN/m at day 2, which continued to decrease until 28 days (One-way ANOVA, p ≤ 0.05).

Liposomes in aqueous dispersions tend to be relatively unstable and these results were consistent with changes that occur to lipids at elevated temperature or over periods of time, e.g. hydrolysis or lipid peroxidation [[39], [40], [41]]. Although antioxidants are present in SLF in small quantities, they are not fully protective and ascorbate may even catalyse the oxidation of phospholipids in solvent systems [42]. Degradation of the phospholipids can reduce pH as lipids dissociate into their constituent fatty acids [43]. Metal cations, such as the sodium ion present in DPPG and the quaternary ammonium ion in DPPC, can participate in reactions that produce a sharp decrease in viscosity [43]. Hydrolysis of phospholipids produces two main lysoforms, with the 1-acyl lysoform being predominant [44]. Higher levels of lipids reduce the surface tension of protein-containing solutions [45] and the electrical conductivity of aqueous solutions [46].

TLC analysis was used to probe lipid stability further. Potassium permanganate was used to detect phospholipids by oxidising the phosphorus groups giving a distinctive colour change from pink to yellow. At day 0, TLC of SLF revealed two spots with RF values of 0.66 ± 0.01 and 0.85 ± 0.01, which were identified as DPPC and DPPG, respectively, by comparison to reference standards (data not shown). From day 3 and day 2, SLF stored at 20 °C and 37 °C exhibited an additional spot with RF value of 0.48 ± 0.01, indicating presence of a lipid hydrolysis product. The lipidomic analysis which would be needed to identify the lipid species is beyond the scope of this study, but TLC was used for quantitative determination [47] using image analysis to provide a semi-quantitative measure of spot intensity for DPPC and DPPG. For SLF stored at 20 °C, DPPC concentration fell by approximately 15% and DPPG concentration fell by 25% by day 3. For SLF stored at 37 °C, both DPPC and DPPG concentrations decreased by approximately 20% by day 2. In contrast, less than 10% loss of DPPC and DPPG was measured in SLF stored at 4 °C.

### 3.4 Recommendations for use of SLF

SLF can be stored in a refrigerator for 2 weeks based on observations that the appearance, pH, viscosity, conductivity and surface tension remained constant for 14 days at 4 °C, and significant changes in SLF characteristic do not become evident until day 28 (One-Way ANOVA, p < 0.05) (Table 2). At 20 °C, changes in the SLF characteristics only became significant after 3–4 days of storage, indicating that SLF is stable for 48 h and can be used in laboratory experiments, e.g. solubility or dissolution tests, carried out at room temperature. At 37 °C, changes in SLF characteristics became significant at day 2, indicating that *in vitro* experiments which involve cell culture or the use of physiological temperature should be restricted to 24 h. Deteriorated SLF can influence *in vitro* assay results, for example FP solubility increases from 2.20 ± 0.29 μg/mL in freshly prepared SLF to 6.80 ± 0.55 μg/mL in SLF stored at 37 °C for 7 days. In contrast, FP solubility was identical in freshly prepared compared to freeze dried and reconstituted SLF.

**Table 2:** The day at which the simulated lung fluid (SLF) changed with regards to its appearance and physicochemical properties (tested at days 1, 2, 3, 4, 7, 14, 28) under storage at 4, 20 and 37 °C.

Physicochemical property	Day at which there is a significant difference^a^ from day 0 [day]		
	4 °C	20 °C	37 °C
Appearance [mean grey value]	28	3	2
pH	28	3	3
Conductivity [mS/m]	28	3	2
Viscosity [Pa.s x 10^−3^]	28	4	3
Surface Tension [mN/m]	28	4	2
**Stability**	**14 days**	**2 days**	**1 day**

a Significance determined by One-way ANOVA, p ≤ 0.05.

**Fig. 3:**
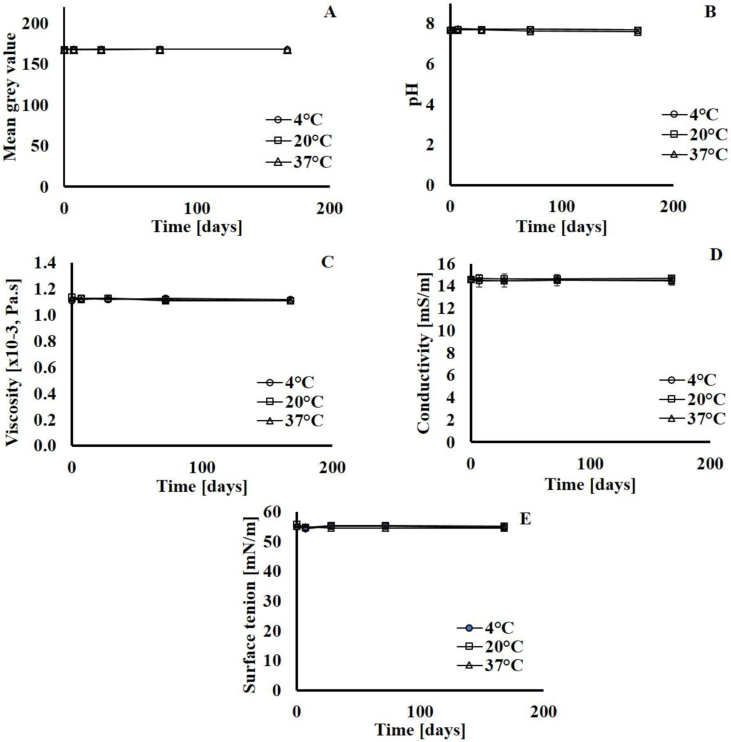
Stability assessment of freeze-dried simulated lung fluid (SLF) after its storage at 4, 20 and 37 °C for 0, 7, 28, 72 and 168 days: A) Colour, B) pH, C) viscosity, D) conductivity, and E) surface tension. Data expressed as mean ± SD (n = 3).

The stability of the lyophilised powder is currently under investigation using the assays described herein. In an ongoing stability study of the lyophilised powder stored at 4, 20 and 37 °C, the appearance, pH, viscosity, conductivity and surface tension in the reconstituted fluid are unchanged after 6 months of storage (Fig. 3) with no chemical changes to the lipids detected by TLC. Thus, improved stability of lyophilised liposomal formulations in contrast to the aqueous formulations and thus allows for a longer-term storage of material. Lyophilisation clearly increases the shelf-life of liposomes, preserving it in a dry form that can easily be reconstituted with water immediately prior to use.

## 4 Conclusion

A SLF of biorelevant composition was characterised and shown to possess physicochemical properties comparable to those of RTLF. The simulant was determined to be stable for 24 and 48 h at 37 and 20 °C, respectively (in-use stability) and for 14 days when stored in a refrigerator (storage stability). Colour, surface tension and conductivity were the most sensitive indicators of product deterioration. The SLF can be freeze-dried which provides a means of prolonged storage. Initial data indicates the stability of the freeze-dried preparation at room temperature for up to 3 months, with studies ongoing. This work describes a readily available, biorelevant SLF that can be used for *in vitro* investigations in the field of inhalation biopharmaceutics, e.g. the solubility of inhaled compounds, the dissolution of inhaled medicines and the interaction of aerosol drugs or particles with lung cells. Experimental *in vitro* data such as this is increasingly in demand to provide inputs to computational models that predict (i) drug bioavailability in the lungs; (ii) the rate and extent of absorption to the systemic circulation [3].

## Conflict of Interest

There are no known conflicts of interest associated with this publication and there has been no significant financial support for this work that could have influenced its outcome.
